# Evaluation of Stoffenmanager and a New Exposure Model for Estimating Occupational Exposure to Styrene in the Fiberglass Reinforced Plastics Lamination Process

**DOI:** 10.3390/ijerph17124486

**Published:** 2020-06-22

**Authors:** Seokwon Lee, Sangjun Choi, Kyoungho Lee

**Affiliations:** 1 Department of Public Health, Graduate School, The Catholic University of Korea, Seoul 06591, Korea; sukwon23@gmail.com; 2 Samsung Health Research Institute, Samsung Electronics Co., Ltd., Hwaseong 18448, Korea; 3 Department of Occupational Health, Daegu Catholic University, Gyeongsan 38430, Korea; junilane@gmail.com

**Keywords:** exposure modelling, fiberglass reinforced plastics (FRP), model evaluation, occupational exposure assessment, Stoffenmanager^®^, styrene

## Abstract

This study aims to evaluate occupational exposure models by comparing model estimations of Stoffenmanager, version 8.2, and exposure scores calculated using a new exposure model with personal exposure measurements for styrene used in the fiberglass-reinforced plastic (FRP) lamination processes in Korea. Using the collected exposure measurements (*n* = 160) with detailed contextual information about the type of process, working conditions, local exhaust ventilation, respiratory protections, and task descriptions, we developed a new model algorithm to estimate the score for occupational exposures on situation level. We assumed that the source of exposure originates from the near field only (within the breathing zone of workers). The new model is designed as a simple formula of multiplying scores for job classification, exposure potential, engineering controls, chemical hazard, and exposure probability and then dividing the score for workplace size. The final score is log-transformed, ranging from 1 to 14, and the exposure category is divided into four ratings: no exposure (1), low (2), medium (3), and high (4) exposures. Using the contextual information, all the parameters and modifying factors are similarly entered into the two models through direct translation and coding processes with expert judgement, and the exposure estimations and scores using the two models are calculated for each situation. Overall bias and precision for Stoffenmanager are −1.00 ± 2.07 (50th) and −0.32 ± 2.32 (90th) for all situations (*n* = 36), indicating that Stoffenmanager slightly underestimated styrene exposures. Pearson’s correlation coefficients are significantly high for Stoffenmanager (*r* = 0.87) and the new model (*r* = 0.88), and the correlation between the two models is significantly high (*r* = 0.93) (*p* < 0.01). Therefore, the model estimations using Stoffenmanager and the new model are significantly correlated with the styrene exposures in the FRP lamination process. Further studies are needed to validate and calibrate the models using a larger number of exposure measurements for various substances in the future.

## 1. Introduction

Under the Registration, Evaluation, Authorisation and Restriction of CHemicals (REACH) Regulation (EC 1907/2006) implemented by the European Chemicals Agency (ECHA), manufacturers or importers are required to conduct a chemical safety assessment, including exposure assessment and risk characterisation, for new or existing chemical substances (≥1 tonne per year), based on the established exposure scenarios for general registration purposes within the European Union [1,2]. Similar to the REACH, the Act on the Registration, Evaluation, etc. of Chemicals (ARECs), known as the Korean version of the REACH, was also enacted and has been enforced since 2015, and this regulation was most recently amended and came into force on 1st January, 2019 [3]. All the registrants who manufacture or import more than 10 tonnes per year are required to conduct a risk assessment and submit the data and relevant information on physical and chemical properties and the risks of chemical substances to the Ministry of Environment, including exposure scenarios describing handling, exposure controls, and appropriate risk management measures (RMMs) during the entire life cycle [4,5]. To comply with European and Korean legislation, occupational exposure assessment using quantitative exposure models can be performed for risk characterisation, assessing the risks of inhalation and dermal exposures to hazardous substances on all tasks, activities and processes, determining adequate control strategies and developing RMMs on the established exposure scenarios in the workplaces when the exposure monitoring data are insufficient or unavailable [3,6].

When conducting exposure assessments to evaluate the magnitude of occupational exposures in particular, two REACH exposure models—ECETOC Targeted Risk Assessment and Stoffenmanager^®^ (hereafter referred to as Stoffenmanager)—have been widely employed and evaluated for the prediction of inhalation exposures to hazardous chemicals in several exposure scenarios in the environment and workplaces in Korea [7,8,9,10]. These studies found that both ECETOC TRA and Stoffenmanager have moderate and high correlations in the estimated exposures compared with measured exposures, and they are also conservative and thus applicable to the given exposure scenarios in Korean workplaces. Lee et al. [11] also conducted a validation study to compare the predicted estimates using three REACH exposure models—ECETOC TRA, Stoffenmanager, and Advanced REACH Tool (ART)—with repeated exposure measurements for solvents in various industries in Korea. They found that Stoffenmanager is the most balanced model with good accuracy, high correlation, and a medium level of conservatism compared to the other models. However, most authors have suggested that further studies would be necessary to evaluate the REACH models by comparing with large-scale exposure measurements, modifying them into the best fitted model by adjusting the model’s original settings for modifying factors, assigned multipliers, and input parameters, especially for PROcess Categories (PROCs), and developing a new exposure model for the applicability domain [9,11].

Recently, there have been several studies over the past few years attempting to develop new and more advanced exposure models for occupational settings, including one-box [12] and two-box dispersion models [13], a Tool for EXposure ASsessment called TEXAS using Bayesian statistics [14], dermal ART [15], welding ART [16], TRanslation of EXposure MOdels (TREXMO) [17], and an advanced exposure model called TREXMO plus incorporated with a machine-learning technique [18]. These studies have reported that new models perform somewhat better than the existing REACH models in the estimation of occupational exposures to various substances, but these models also had uncertainties and variabilities in input parameters and model predictions with low levels of accuracy, precision, and reliability, similarly observed in the REACH models. Thus, most authors have recommended that further validation and calibration using large-scale exposure measurements still be required for future refinement of new models and cutting-edge approaches for a large number of substances in different exposure situations and scenarios in Western countries.

Styrene is used as the raw material (as monomer and in copolymers) in the production of various plastic and rubber products, including polystyrene, acrylonitrile butadiene styrene, styrene butadiene rubber, unsaturated polyester resins (UPR), and other resins [19]. The health effects of styrene are mainly associated with lymphohaematopoietic malignancies, neurotoxicity, and respiratory and allergic diseases, and the overall evaluation for styrene is classified as probably carcinogenic to humans (Group 2A) [20]. In occupational settings, the highest exposure to styrene occurs in the reinforced plastics industry [21,22], but exposure levels have apparently decreased over the past years as a result of the improvement in workplaces and practices [23]. In Korea, styrene has widely been used in the FRP lamination, coating, and moulding processes in plastic, chemical, or underground storage tank (UST) manufacturing, ship building, automotive products and repair parts, and other manufacturing industries since 1990s, and a large number of workers were more highly exposed to styrene during UPR spraying, coating, and packaging tasks in the FRP lamination process than in other processes [24]. Recently, the overall risks of occupational exposure to styrene in various workplaces and industries in Korea were also evaluated using a large-scale exposure measurement dataset collected by KOSHA (*n* > 9000), and the FRP lamination process in the plastic manufacturing industry appeared to post the highest risk for styrene exposure [25]. However, the levels of styrene exposure in the FRP lamination process were not significantly different among various types of industries classified by Korean standard industrial classification, but they differed by type of subtask and engineering control [26]. A case study was conducted to evaluate occupational exposure to styrene used in the FRP lamination process in a double-walled UST manufacturing company in 2008 [27]. The authors identified that workers performed spraying, coating, and rolling tasks for UST manufacturing, as well as visual inspection tasks for quality control after completing manufacturing. Based on these study findings, exposure profiles have been defined and classified into two similar exposure groups (SEGs) of workers performing the following tasks: (1) industrial spraying and coating using UPR; and (2) inspection for quality control in Korean workplaces.

To date, however, no studies have been performed to develop a new exposure model specialised for the prediction of occupational exposures to chemical substances in task-specific exposure situations and to further evaluate its accuracy, reliability and applicability by comparing it with the REACH models and real-world exposure measurements in Korea. Furthermore, there remain uncertainties in the model inputs and estimated outcomes due to a lack of information about the types of processes, task descriptions, working conditions, general ventilation, control measures, and respiratory protective equipment in the given exposure scenarios. Most importantly, there has been little evidence on which exposure models would be the most accurate and least biased, as well as which information should be collated to assess the magnitude of occupational exposures in Korean workplaces within the scientific community in recent years.

Therefore, this study aimed to develop a new semi-quantitative model enabling the calculation of exposure scores and evaluating occupational exposure models by comparing the model estimations using Stoffenmanager and the new model with historical exposure measurements for styrene in the exposure situations, such as industrial spraying, coating and inspection tasks, in the fiberglass-reinforced plastic (FRP) lamination processes in Korea.

## 2. Materials and Methods

### 2.1. Data Collection

We collected historical exposure measurements of styrene used for the manufacturing of ships and boats, automotive products and parts, double-walled UST, and sluice in the FRP lamination processes at 22 small- and medium-sized companies located in different provinces in Korea during 2003–2010 [26,27]. The exposure measurement datasets included all personal samples collected for time-weighted average (8h-TWA) concentrations in parts per million (ppm), quantified using National Institute for Occupational Safety and Health (NIOSH) method #1501. Detailed contextual information about various exposure determinants, including type of process, environmental condition (process temperature, relative humidity), process operation (manual or semi-automated), task description (frequency and duration), number of workers, use of local exhaust ventilation (LEV), general ventilation and containment, respiratory protective equipment (RPE), quantity of styrene used (kg/month), percentage of styrene in the UPR products, and current Korean occupational exposure limit (KOEL), was also collected from each company by means of a review process of written documents provided and on-site interviews with workers and EHS managers. Exposure assessment was conducted by Korean industrial hygienists affiliated with several private consulting companies, specialising in industrial hygiene (IH) monitoring practices and accredited by the Ministry of Employment and Labour (MoEL) in Korea.

Furthermore, we also collected a total of 541 repeated exposure measurements for 11 chemical substances, including styrene [28], in various situations, including manufacturing, industrial spraying, coating, mixing, solvent cleaning, and packaging tasks, at 45 different companies and 20 industries in the mid-2000s from MoEL survey reports, collected and employed in a previous study for research on the feasibility of the generic applicability of three REACH models in Korean workplaces [11]. The collected exposure measurements and detailed contextual information were used as the reference information for the development of a new model in which determining parameters and modifying factors, including position factor, potential emission and handling, type of equipment, local ventilation and containment, control measures, personal protective equipment, task frequency and duration, distance from the source, and work room volume (m^3^), as well as to adequately select certain numeric scores for defined classification of each modifying factor.

### 2.2. Development of a New Semi-Quantitative Exposure Model

We developed a new semi-quantitative exposure model that enables the calculation of a log-transformed exposure score for each exposure situation or job task and to determine one of four exposure categories depending on the calculated scores. The new model consists of a simple algorithm to estimate inhalation exposure to hazardous chemicals emitted from the near-field (NF) source in indoor workplaces. The new model is essentially similar to several existing models, such as structured subjective assessment [29], a quantitative model algorithm for pesticide exposure assessment [30], ECETOC TRA [31,32], Stoffenmanager [33,34], and ART [35,36,37], previously developed and validated in European countries over several decades. Most of the authors employed a two-box model for development of exposure estimation models under the assumption that the emissions of air contaminants are fully evaporated from various sources and well mixed in the workroom, subdivided into two compartments with the first box (NF zone) located within a distance of 1 m from the breathing zone of a worker during job tasks and the second box (far-field zone, FF) indicating the remainder area of the working environment (indoor or outdoor).

In real-world workplaces, however, IH monitoring data are generally collected either within the breathing zone of workers using a personal sampler (active or passive) or near the fixed location where the workers spend the most time while a task is performed, and the personal exposure monitoring method has been accepted and used for the purpose of regulatory exposure assessment in workplaces to comply with the Occupational Safety and Health Act in Korea since 1998 [38]. In this regard, we assume that the occupational exposures to chemicals mostly came from NF sources, and background and secondary exposures to volatile chemicals in different situations and job tasks performed in the same workplaces are ignored and thus excluded from the new model algorithm. Furthermore, it is considered that neither dermal nor ingestion exposure occurs, not only because all the exposure measurements were collected only for inhalation exposure to various chemicals among workers in various manufacturing industries but also because the distributions of occupational exposures were significantly influenced by the use of engineering controls, such as LEV, RPE, etc. Therefore, the new model is similar to a semi-quantitative risk assessment tool for the evaluation of inhalation exposure to chemicals mainly emitted from the NF sources at workplaces, rather than a two-box exposure model used in the mechanistic REACH models. In this regard, we designed and developed the new model for simple but more realistic assessment and estimation of the average exposure to air contaminants emitted from a point source in a large work room of an indoor or outdoor environment.

Twelve modifying factors, including position factor (*P_f_*), potential emission (*E_p_*), historical exposure (*E_h_*), type of process with tool cleaning, inspection and maintenance (*E_m_*), ventilation and containment (*η_gv_*), localised controls (*L_c_*), personal protective equipment (*PPE*), health hazard category (*H*), task duration (*T_h_*), task frequency (*T_f_*), distance from the source (*D*), and room volume (*V*), are included and reflected in this model. The new model algorithm consists of a simple formula of multiplying numeric scores for 11 modifying factors and dividing by a score of room volume, and then the calculated exposure scores were finally log-transformed using Equation (1) as follows.

(1) $$\mathrm{Exposure}\;\mathrm{score}=ln\;\left[\frac{P_{f}\times \left(\;E_{p}\times E_{h}\times E_{m}\right)\times \left(\eta_{gv}\times L_{c}\times PPE\right)\times H\times \left(T_{h}\times T_{f}\times D\right)}{V}\right]$$

The quantitative and qualitative (contextual) information about exposure determinants, input parameters and factors was acquired from historical exposure monitoring data, previous IH survey reports, visual observations during site visits, and on-site interviews with workers performing various job tasks using chemical substances. Using the collated information, each score of 12 modifying factors is assigned into one of the defined classifications with reference to several previous studies, mainly aiming to develop the REACH models [29,33,35] and the other models [30,39], as well as to quantitatively evaluate model accuracy, lack of agreement, and correlations with Korean exposure measurements [11]. We assumed that a score of 1.0 is the lowest score for each modifying factor, indicating that a task is related to a situation of “no exposure (office worker)”; thus, the default outcome of final exposure score is set to 1.0. We also assumed that a job task is regularly performed 8 h per day and for 5 working days per week when calculating the final exposure scores using this new model.

More specifically, we applied similar classifications and categories for principal modifying factors previously used in both Stoffenmanager and ART models [29,33], but we selected different scores for each classification based on differences in the overall level of exposures to liquid and solid substances in the new model algorithm. For example, an average level of exposure on the tasks without using LEV is approximately three times higher than the level of exposure using LEV. Similarly, the mean exposure levels with no PPE are five times higher than the level of worker protection using PPE (>90%) during tasks. In addition, the levels of occupational exposure to volatile liquids in the same situation or task also increased 1.7 times more in manual operations than in fully automated process operations.

All the defined classifications of the modifying factors and associated scores were internally peer reviewed and determined by three IH professionals participating in the present study. We described detailed information about the types of input parameters and modifying factors, defined classifications and associated scores for the newly developed model with relevant references (Table 1). The calculated final score for each situation was log-transformed, ranging from 1 to 14, and an exposure category was classified into one of the four ratings―no exposure (1), low (2), medium (3), and high (4) exposures―depending on exposure profiles (Table 2).

### 2.3. Data Input and Parameter Translation

We followed the same practices for the review and translation of contextual information about exposure determinants and situations, as well as determination of the most appropriate parameters and modifying factors in Stoffenmanager, version 8.2 (https://stoffenmanager.nl/), and the new exposure model, as similarly described in a previous study [11]. Whereas a total of 20 modifying factors, including a parameter of PROcess Category (PROC), which is newly available to select in the Stoffenmanager model, were also entered to predict inhalation exposure to styrene in Stoffenmanager, 17 modifying factors were entered into the new model. Table 3 shows the examples of parameters and modifying factors entered in both Stoffenmanager and the new model. Most of the parameters and modifying factors are directly translated and coded from the collected information about exposure situations and job tasks, but some factors, for example, position factor, type of subtask, PROC, ventilation, control measures, and inspection and maintenance of equipment, were entered into the models using expert judgements for the prediction of model estimations and exposure scores on situation level. All the parameters and modifying factors entered in each model are provided in the Supplementary Materials, available in the *International Journal of Environmental Research and Public Health*. The default outcomes in Stoffenmanager are set as the 50th and 90th percentiles of the distributions of inhalation exposure to styrene for all situations.

### 2.4. Evaluation of Stoffenmanager and the New Model

We used the same approaches for model evaluation [11]. First, lack of agreement (bias, relative bias, and precision) was calculated using Equations (2)–(4) as follows.

(2) $$\mathrm{Bias}=\sum_{i=1}^{n_{o}}\frac{\left({\hat{y}}_{i}-y_{i}\right)}{n_{0}}$$

(3) $$\mathrm{Relative}\;\mathrm{bias}=\left(e^{bias}-1\right)\times 100\%$$

(4) $$\mathrm{Precision}=\sqrt{\overset{n_{o}}{\underset{i=1}{\sum }}\frac{{\left[\left({\hat{y}}_{i}-y_{i}\right)-bias\right]}^{2}}{n_{0}-1}}$$

where: ${\hat{y}}_{i}=ln\;\left(\mathrm{modeled}\;\mathrm{estimates}\;\mathrm{for}\;\mathrm{the}\;i\;\mathrm{th}\;\mathrm{set}\;\mathrm{of}\;\mathrm{exposure}\;\mathrm{in}\;\mathrm{the}\;\mathrm{validation}\;\mathrm{dataset}\right)$;

$y_{i}=ln\;\left(\mathrm{measured}\;\mathrm{exposures}\;\mathrm{for}\;\mathrm{the}\;i\;\mathrm{th}\;\mathrm{set}\;\mathrm{of}\;\mathrm{exposure}\;\mathrm{in}\;\mathrm{the}\;\mathrm{measured}\;\mathrm{dataset}\right)$

$n_{0}=\mathrm{number}\;\mathrm{of}\;\mathrm{measurements}\;\mathrm{in}\;\mathrm{the}\;\mathrm{validation}\;\mathrm{dataset}$

We also calculated accuracy and mean absolute error (MAE), using Equation (5), which implies the average distance between the estimated and measured exposures [18], and we used the 50th and 90th percentiles in Stoffenmanager to compare with exposure measurements.

(5) $$\mathrm{Accuracy}=\overset{n_{o}}{\underset{i=1}{\sum }}\frac{\left|{\hat{y}}_{i}-y_{i}\right|}{n_{0}}$$

Second, residuals, which are the differences between the log-transformed modelled estimates and measured exposures, were also calculated using Equation (6) as follows.

(6) $$\mathrm{Residual}={\hat{y}}_{i}-y_{i}$$

The daily average or 90th percentiles of the estimated exposure distributions in Stoffenmanager were used to calculate the log differences with historical exposure measurements. We demonstrated the residual plots showing the mean differences with upper and lower limits of agreement on individual level. Lack of agreement (bias, relative bias, precision, and accuracy) and residuals were calculated only for the Stoffenmanager estimations.

Third, we calculated Pearson’s correlation coefficients (*r*) between the log-transformed model outcomes of Stoffenmanager (90th percentile) and the new model (final exposure score) and historical exposure measurements, and we also demonstrated scatterplots with a fitted linear line with corresponding 95% confidence intervals (CIs).

### 2.5. Statistical Analysis

We used STATA software, version 12.1 (StataCorp LP, College Station, TX, USA), for all data analyses. All the individual model estimations and exposure measurements were log-transformed before calculating the descriptive statistics, lack of agreement, residuals, and Pearson’s correlation coefficients. We also performed Anderson-Darling normality testing to see whether all the data are log-normally distributed at a significance level of 0.05.

## 3. Results

Table 4 presents descriptive statistics of historical exposure measurements collected for styrene (*n* = 160) during the FRP lamination (industrial spraying) and inspection tasks by company. 49 of 160 exposure measurements exceeded 20 ppm of KOEL (8h-TWA) (31%). The GMs (GSD) were 13.07 (2.86) for the FRP lamination (*n* = 137) and 0.23 (4.25) for inspection situations (*n* = 23). 

**Figure 1:**
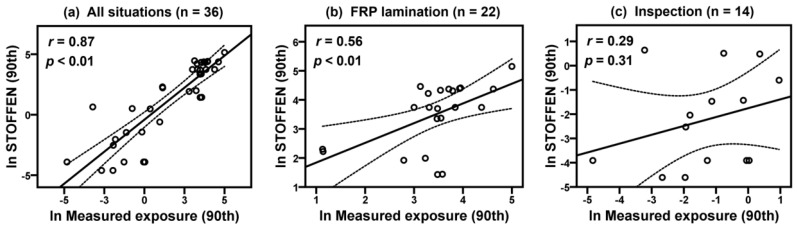
Scatterplots of the log-transformed model estimations (90th) of Stoffenmanager and exposure measurements (90th) for styrene collected on the FRP lamination and inspection tasks on situation level. The dashed lines indicate corresponding 95% confidence intervals (CIs).

**Figure 2:**
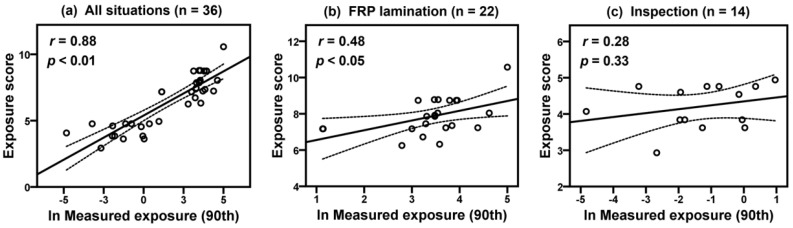
Scatterplots of exposure scores calculated using a new model and log-transformed exposure measurements (90th) for styrene collected on the FRP lamination and inspection tasks on situation level. The dashed lines indicate corresponding 95% CIs.

**Figure 3:**
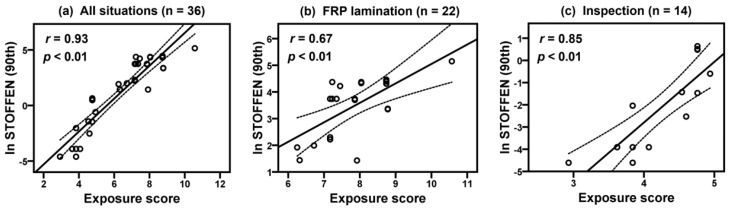
Scatterplots of the log-transformed model estimations (90th) of Stoffenmanager and exposure scores calculated using a new model for styrene collected on the FRP lamination and inspection tasks on situation level. The dashed lines indicate corresponding 95% CIs.

**Figure 4:**
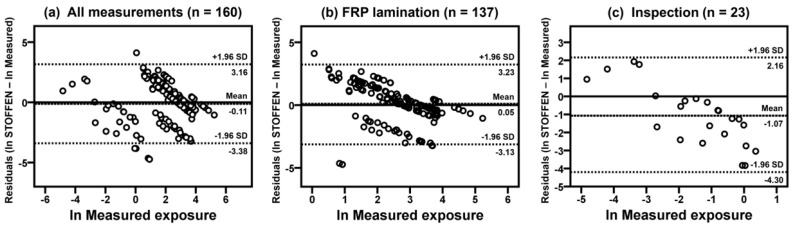
Residual plots of the differences between the log-transformed model estimations (daily average or 90th) of Stoffenmanager and exposure measurements for styrene collected on the FRP lamination and inspection tasks on individual level. The mean differences with 95% upper and lower limits of agreement are marked with the dashed lines.

Table 5 shows the results of comparison of model estimations (daily average, 50th and 90th percentiles in Stoffenmanager and exposure scores of the new model) and historical exposure measurements (GM and 90th) of each company. The lack of agreement is calculated to compare the 50th and 90th model estimations of Stoffenmanager with the exposure measurements (Table 6). Overall bias and precision were −1.00 ± 2.07 (50th) and −0.32 ± 2.32 (90th), respectively, and relative bias was −63.32% (50th) and −27.30% (90th), indicating that Stoffenmanager underestimated styrene exposure levels. Overall accuracy was calculated as 1.28 (50th) and 1.16 (90th) for the Stoffenmanager estimations. Figure 1 shows correlations between the log-transformed 90th percentile estimates of Stoffenmanager and 90th percentiles of exposure measurements with Pearson’s correlation coefficients (*r*) and *p*-values for all situations (*r* = 0.87, *p* < 0.01), FRP lamination (*r* = 0.56, *p* < 0.01), and inspection (*r* = 0.29, *p* = 0.31). Figure 2 presents correlations between exposure scores using the new model and 90th exposure measurements in all situations (*r* = 0.88, *p* < 0.01), FRP lamination (*r* = 0.48, *p* < 0.05), and inspection (*r* = 0.28, *p* = 0.33). Figure 3 shows that correlations between the 90th percentile estimates of Stoffenmanager and calculated exposure scores using the new model are highly significant for all situations (*r* = 0.93, *p* < 0.01), FRP lamination (*r* = 0.67, *p* < 0.01), and inspection (*r* = 0.85, *p* < 0.01). Residual plots of the differences between the Stoffenmanager estimations and individual measurements are shown in Figure 4. The overall mean differences are −0.11 with 95% upper (3.16) and lower (−3.38) limits of agreement. In the residual plots, a systematic tendency to overestimate low exposures and underestimate high exposures was also observed.

## 4. Discussion

In this study, we characterised styrene exposure levels collected in industrial spraying and coating situations in the FRP lamination process in various industries in Korea, sometimes exceeding 20 ppm which is the current KOEL, but they were relatively low in the inspection situation. The Stoffenmanager estimations and log-transformed exposure scores calculated by the new model were significantly correlated with historical exposure data with good accuracy. Moreover, the correlations between the estimated outcomes of these two models were highly significant for all situations, suggesting potential applicability of both models to Korean workplaces. As we anticipated, Stoffenmanager, one of the high Tier REACH models, accurately estimated inhalation exposure to styrene for the given situations in the FRP lamination process, but the model slightly underestimated the styrene exposures. We also observed a systematic tendency to overestimate low exposures and underestimate high exposures in Stoffenmanager, as similarly shown in many studies [11,45,46,47,48,49,50,51]. Despite the uncertainty, the overall performance of Stoffenmanager was good, accurate, and less biased (close to zero), and the newly developed model was also capable of evaluating the magnitude of styrene exposures for the industrial spraying situation. Therefore, our study results suggest that Stoffenmanager can be used as a generic model for estimating occupational exposures based on the model performance, reliability, and consistency. More importantly, the new model’s outcomes, similarly designed as a risk assessment framework, were comparable to the Stoffenmanager estimations, although we employed a relatively small number of historical exposure data and employed different parameters and modifying factors with the new model algorithm based on contextual information about exposure determinants and factors mainly collected from real-world workplaces in Korea.

To our knowledge, we have developed for the first time a simple, convenient, and universal model of estimating chemical exposure scores specialised for Korean situations by collecting the minimum necessary information about exposure determinants and assessment factors affecting the exposure levels, including process automation, type of equipment, local exhaust ventilation, respiratory protections, and task descriptions. We also selected the most suitable input parameters and modifying factors with scores in the new model with reference to principal modifying factors in two high Tier REACH models, Stoffenmanager and ART, but each score for the classified modifying factors was determined by the differences in actual exposure levels previously measured in various workplaces and industries during chemical tasks performed in Korea in the mid-2000s. Furthermore, the same assessment factors and associated scores for some parameters, such as position factor, historical exposure, personal protective equipment, and health hazard category, were employed as the existing risk assessment tools, including the COSHH Essentials [43], quantitative pesticide exposure algorithm [30], qualitative exposure assessment (QLEA) model [41], Chemical HAzard Risk Management (CHARM) [42], and chemical exposure algorithm [39], and were directly applied to our new model algorithms without modification.

The newly developed model is basically intended to support EHS managers, IH professionals, and individual field workers in identifying health hazards, evaluating occupational exposures, and characterising and controlling the potential risks of chemical exposures in Korean workplaces, similar to the framework of risk assessment tools. Using the same exposure data and contextual information, Kim and Choi [52] previously conducted a study comparing the outcomes using two risk assessment tools, COSHH Essentials and CHARM, for styrene exposures in the FRP lamination process in Korea. The authors found that the exposure and hazard bands are 3 and B, respectively, and they concluded that all the final risk levels (control strategy) are 2 (a medium level of exposure risk recommending the use of engineering controls), which is exactly identical to our new model’s outcomes, and the exposure category is 3 (a medium level of styrene exposures). Therefore, our new model can be used as a generic risk assessment tool for determining a categorical rating for occupational exposure risk, but it is more likely to be a very early edition of the exposure model to estimate and evaluate the magnitude of occupational exposures to chemical substances specific to the Korean situations when the measured exposure is unavailable.

In previous studies, many authors began by developing new semi-quantitative or qualitative modelling tools to calculate numeric scores for assessment, characterisation and control of occupational exposures and associated health risks regarding hazardous substances at the initial stage, and then they further calibrated, validated, and refined their developed models into advanced quantitative models to more accurately predict the point estimates of exposure distributions using large-scale exposure data in the next step. In the Netherlands, Marquart et al. [33] developed a new web-based risk banding scheme, called Stoffenmanager, to calculate numeric scores for occupational exposure to various hazardous substances in small- and medium-sized enterprises by combining the hazard banding of COSHH essentials and exposure banding from a new model developed by Cherrie and Schneider [29]. The subsequent studies [34,45,46] reported good accuracy, medium conservatism, and reliability with Stoffenmanager after precisely fitting and incorporating the model outcomes (log-transformed exposure scores) with the measured exposures for solid and liquid scenarios using mixed-effects regression models. Therefore, the overall model performance of Stoffenmanager was good enough to be employed as a generic modelling tool for occupational exposure assessment in the worst-case scenarios despite some uncertainties.

A few years later, a more refined mechanistic model, called as ART, based on a conceptual source-receptor framework, was also designed and developed to provide more realistic estimates of occupational exposure to vapours, mists, and dust in the reasonable worst-case scenarios employing Bayesian statistics [35,36,37]. Like Stoffenmanager, Schinkel et al. [53] also calibrated the ART model using large-scale exposure data (*n* > 2000) for various scenarios and situations, and it was further upgraded and modified into a better version (1.5) by incorporating larger exposure data from 117 different situations [54]. Since then, ART has been widely evaluated by comparing the modelled estimations with different exposure measurements collected from various workplaces and industries in many countries [11,47,48,49,51,55,56]. Other REACH models, such as Stoffenmanager nano-objects [57,58], weld ART [16,59], and dermal ART [15,60], have also been developed, calibrated, and validated by applying the same procedures, activities, and approaches. Compared with the early versions of REACH models developed at the initial stage, our new model showed quite accurate outcomes with high correlations with historical exposure data and consistency with the Stoffenmanager estimations and risk assessment outcomes.

However, our new model significantly differs from the existing REACH models for the two following reasons. First, the mechanistic REACH models were originally designed and developed from a conceptual source-receptor exposure model, and detailed information about the intrinsic emission potential of substances with process temperatures, molecular weights, vapour pressures, and mole fractions of components is important and thus entered into the REACH models but was not considered in our new model. Moreover, a factor of PROC was newly included as one of the important modifying factors in the latest version of Stoffenmanager (8.2). We entered PROC 7 (industrial spraying) to estimate the styrene exposure situations in Stoffenmanager, but they were not entered into our new model. These two factors, intrinsic emission potential and a factor of PROC, are well known to be the most important determinant factors significantly affecting the modelled outcomes, and the selection of different parameters or modifying factors for the same scenarios can sometimes produce random uncertainties [47]. Second, numeric scores for the categorised classifications of modifying factors were determined by the differences in actual chemical exposure levels calculated using historical exposure data with contextual information collected from Korean situations. The exposure score for each classified modifying factor is between 1.0 and 10 in our new model, whereas the range of assigned multipliers in the REACH models is from 0.0001 to 30, presenting a difference between the models. Furthermore, a score of 1.0 was set as the default value for each score and the calculated final exposure score, indicating no exposure (office worker), whereas the REACH models set a score of 1.0 as a certain exposure concentration for a given scenario or situation. In this regard, these differences led to completely different modelled outcomes in Stoffenmanager (0.10–173.02 ppm) and the new model (2.93–10.57 score) in this study. Despite the differences, the new model’s outcomes were significantly correlated with the Stoffenmanager estimations and historical exposure measurements, and we were able to accurately evaluate styrene exposure levels using the new model by situation (low on inspection and higher on the industrial spraying situations).

This study has limitations. First, the number of historical exposure data used for model evaluation and selection of the input parameters, factors, and assigned scores was relatively small. We were unable to collect a sufficiently large number of exposure measurement data for various chemicals regarding different processes, tasks, workplaces, and industries in Korea. Although we presented high correlations, good accuracy, and consistency in the model outcomes, our results remained limited and not fully validated for other scenarios or situations in Korea. Second, we assumed that no exposure came from the FF sources or backgrounds thus ignored, and neither dermal nor ingestion exposure occurred for the given situations. Under assumptions, we determined the modifying factors and associated scores in the new model based on historical exposure data and contextual information collected from Korean workplaces. If the exposure data and information were incorrect or biased, the model outcomes might have led to under- or overestimation (towards either direction). However, we, including the authors, carefully reviewed and cross-checked all the collected data and information about styrene exposures and confirmed that there was no missing or incomplete information in the datasets. Using the same data and information, three studies were previously conducted to evaluate styrene exposures in the FRP lamination process using different approaches [26,27,52]. There appeared similarity and consistency in all the study results, and uncertainty from the information bias or deficiency could be effectively controlled in this study.

## 5. Conclusions

In summary, we designed and developed an early version of a new semi-quantitative model to evaluate and estimate occupational styrene exposures in the FRP lamination process in Korea. Stoffenmanager, a high Tier REACH model, showed good accuracy and reliability in the model estimations for the given situations. More importantly, the correlations between the two models were highly significant for all situations, suggesting generic applicability of the models to Korean workplaces. However, the new model should be further calibrated and refined into an advanced quantitative model to predict the point estimates of chemical exposures in different scenarios or situations using a larger number of exposure measurement data. Therefore, further studies are needed to more deeply evaluate, validate, and confirm the new model’s performance and reliability for other chemical substances used in various job tasks, processes, workplaces, and industries in Korea in the future.

## Figures and Tables

**Table 1:** A new exposure model for occupational exposure assessment with input parameters, modifying factors, defined classification, and associated scores.

Parameter	Modifying Factor		Classification	Score	Reference
Job classification (= P_f_)	Position factor (P_f_)		Office worker	1.0	Fleming et al. (2014) [39]
			Utility engineer, EHS manager, field inspector, engineer/scientist, supervisor, etc.	1.2	
			General worker (e.g., manufacturing, assembly, quality controls, process operation, etc.)	2.0	
			Maintenance engineer (e.g., solvent cleaning, maintenance of equipment tools, etc.)	3.0	
Exposure potential (= E_P_ × E_h_ × E_m_)	Potential emission and handling of products (E_p_) (either liquid or solid)	Liquid	Neither potential emission nor handling	1.0	Cherrie and Schneider (1999) [29] Marquart et al. (2008) [33] Fransman et al. (2011) [35] Van Tongeren et al. (2011) [37]
			Small amount of chemicals (in grams) is used and may be released	1.5	
			Medium amount of chemicals (in kilograms) is used with low pressure and speed on medium-sized surfaces and workplaces	2.0	
			Large amount of chemicals (in tons) is used with high pressure and speed resulting in generation of mist or spray/haze on large surfaces and workplaces	3.0	
			Extremely large amount of chemicals is used with extremely high pressure and speed resulting in highly substantial generation of mist or spray/haze in workplaces	5.0	
		Solid	Neither potential emission nor handling	1.0	
			Small amount of chemicals (in grams) is used and may be released (little dusty)	1.5	
			Medium amount of chemicals (in kilograms) is used with low pressure and speed on medium or large surfaces or workplaces (some dusty)	2.0	
			Large amount of chemicals (in tons) is used with high pressure and speed resulting in large quantities of dusts generated and dispersed at workplaces (dusty)	3.0	
			Extremely large amount of chemicals is used with extremely high pressure and speed resulting in extremely high quantities of dusts generated and dispersed (very dusty)	5.0	
	Historical exposure (E_h_) ^1^	With IH data	No exposure (either ‘non-detectable’ or ‘below detection limits’)	1.0	AIHA (2007) [40] Elliott et al. (2007) [41] KOSHA (2012) [42]
			Samples were < 10% of exposure limits (e.g., 8h-TWA, STEL, etc.)	1.2	
			Samples were 10–50% of exposure limits	2.0	
			Samples were 50–100% of exposure limits (under the exposure limits)	3.0	
			Samples exceeded the exposure limits (>100%)	5.0	
		No IH data	No exposure	1.0	
			Low level of exposure (fully enclosed and highly controlled)	1.2	
			Medium level of exposure (partially enclosed and well controlled)	2.0	
			High level of exposure (trivially enclosed and slightly controlled)	3.0	
			Extremely high level of exposure (neither enclosed nor controlled)	5.0	
	Type of process with tool cleaning, inspection and maintenance (E_m_)		No exposure	1.0	Marquart et al. (2008) [33] Fransman et al. (2011) [35]
			Full automation of process with daily cleaning, inspection, and maintenance	1.2	
			Semi-automation of process with weekly cleaning, inspection, and maintenance	1.5	
			Manual handling in process with no cleaning, inspection, and maintenance	2.0	
Engineering controls (= η_gv_ × L_c_ × PPE)	Ventilation and containment (η_gv_)		No exposure	1.0	Marquart et al. (2008) [33] Elliott et al. (2007) [41]
			Fully ventilated with high-level containment at the source	1.2	
			Partially ventilated with medium-level containment at the source	1.5	
			General ventilation with low-level containment at the source	2.0	
			Neither ventilation nor containment is used at the source	3.0	
	Localized control measure (L_c_)		No exposure	1.0	Marquart et al. (2008) [33] Fransman et al. (2011) [35] Elliott et al. (2007) [41]
			Local exhaust ventilation (LEV) is used, and all contaminants are fully removed	1.5	
			LEV is used, but some contaminants are not removed and remain	2.0	
			No LEV is used (in the open process) thus no removal of contaminants	3.0	
	Personal protective equipment (PPE)		No exposure	1.0	Dosemeci et al. (2002) [30]
			90% protection using PPE-1 ^2^ and PPE-2 ^3^ and PPE-3 ^4^	1.2	
			70% protection using PPE-2 and PPE-3	1.5	
			50% protection using PPE-1 and PPE-2	2.0	
			40% protection using PPE-3	2.5	
			30% protection using PPE-2	3.0	
			20% protection using PPE-1	4.0	
			0% protection (no use of PPE)	5.0	
Chemical hazard (= H)	Health hazard category (H) ^5^		No exposure	1.0	AIHA (2007) [40] KOSHA (2012) [42] HSE (1999) [43] United Nations (2019) [44]
			Hazard category 4 or 5 (e.g., H302, H303, H312, H332, H333, etc.)	2.0	
			Hazard category 3 (e.g., H301, H311, H316, H331, H335, H336, etc.)	3.0	
			Hazard category 2 (e.g., H305, H315, H319, H320, H371, H373, etc.)	4.0	
			Hazard category 1 or CMR (carcinogenicity, reproductive, or mutagenicity) (e.g., H300, H330, H334, H340, H341, H350, H351, H360, H361, H370, etc.)	6.0	
Exposure probability (= T_h_ × T_f_ × D)	Task duration (T_h_)		No exposure	1.0	Marquart et al. (2008) [33]
			<1 h	1.5	
			1–4 h	2.0	
			4–8 h	3.0	
	Task frequency (T_f_)		No exposure	1.0	Marquart et al. (2008) [33]
			1 day a year	1.2	
			1 day a month	1.5	
			2–3 days a week	2.0	
			4–5 days a week	3.0	
			All-time	5.0	
	Distance from the source (D)		No exposure	1.0	Cherrie and Schneider (1999) [29] Fransman et al. (2011) [35]
			>1 m	1.5	
			≤1 m	2.0	
Workplace size (= V)	Room volume (V)		No exposure	1.0	Marquart et al. (2008) [33]
			<100 m^3^	1.0	
			100–1000 m^3^	5.0	
			>1000 m^3^	10.0	

^1^ Expert judgement can be used to determine a score for historical exposure when industrial hygiene (IH) monitoring data is unavailable; ^2^ PPE-1 (20% protection): Face shields or goggles, dust mask, fabric gloves/apron, and boots; ^3^ PPE-2 (30% protection): Half-face cartridge respirator/gas mask and filters, disposable outer clothing, and protective gloves/apron/boots; ^4^ PPE-3 (40% protection): Full-face cartridge respirator/gas mask and filters, chemical-resistant hooded coverall suit/gloves/boots, and safety helmet; ^5^ Globally Harmonized System of Classification and Labelling of Chemicals (GHS) (United Nations, 2019) was used for classification and determination of health hazard categories (H-codes).

**Table 2:** The classified exposure category and associated descriptions depending on the range of exposure scores and percentages.

Exposure Category ^1^	Description	Range of Exposure Score	Percentage of Exposure Score
1	No exposure (e.g., office worker)	1.00	-
2	Low level of exposure (fully enclosed and highly controlled)	1.01–4.76	<34%
3	Medium level of exposure (partially enclosed and well controlled)	4.77–9.38	34–67%
4	High level of exposure (little enclosed and poorly controlled)	9.39–14.01	67–100%

^1^ Exposure category can be assigned to each exposure situation, job task, or an individual worker based on exposure profile.

**Table 3:** Examples of modifying factors and associated scores entered in Stoffenmanager and a new model with the level of translation using expert judgment in this study.

Modifying Factor	Stoffenmanager	New Exposure Model		Level of Translation
Exposure Situation (Task)	FRP Lamination for Ship Manufacturing	FRP Lamination for Ship Manufacturing	(Score)	
Intrinsic emission source				
Name of agent	Styrene	Styrene	-	Direct translation (Korean → English)
Type of agent	Liquid	Liquid	-	Direct translation
Process temperature	25 °C	-	-	Direct coding
Molecular weight	104.15 g/mol	-	-	Direct coding (Reference.: PubChem)
Vapor pressure	853 Pa	-	-	Direct coding (Reference.: PubChem)
Percentage in product	33–44%	-	-	Direct coding
Job classification				
Position factor	-	General worker (manufacturing, assembly, etc.)	(2.0)	Direct translation with expert judgment
Process				
Type of task (Activity)	Handling of liquids on large surfaces or large workpieces	Large amount of chemicals (in tons) is used with high pressure and speed resulting in generation of mist or spray/haze on large surfaces and workplaces	(3.0)	Direct translation with expert judgment (used the information on type of process, task description, quantity of chemical, etc.)
Historical exposure	-	Samples were 10–50% of exposure limits	(2.0)	Direct translation
Process category (PROC)	PROC7: Industrial spraying	-	-	Direct translation with expert judgment
Task duration	180 min	1–4 h	(2.0)	Direct coding
Task frequency	4–5 days a week	4–5 days a week	(3.0)	Direct coding
Distance from the source	Yes (≤1 m, in breathing zone)	≤1 m	(2.0)	Direct coding
Coworker carrying out the same task	Yes	-	-	Direct coding
Evaporation, drying, or curing	Yes	-	-	Direct translation
RPE	Reusable half mask respirator-gas/vapour filter	50% protection using PPE-1 and PPE-2	(2.0)	Direct translation with expert judgment
Chemical hazard				
Health hazard category	-	Hazard category 2 (e.g., H305, H315, H319, etc.)	(4.0)	Direct coding (Reference.: PubChem)
Workplace				
Room volume	>1000 m^3^	>1000 m^3^	(10.0)	Direct coding
General ventilation	Good ventilation (open windows and doors)	Fully ventilated with high-level containment	(1.2)	Direct translation with expert judgment
Control measure	Local exhaust ventilation (LEV)	LEV is used, but some contaminants are not removed and remain	(2.0)	Direct translation with expert judgment
Inspections and maintenance of equipment	Yes	Manual handling with low-level cleaning	(2.0)	Direct translation with expert judgment
General house cleaning	Yes	-	-	Direct translation
Protection of employee	Not work in a cabin	-	-	Direct translation

**Table 4:** Descriptive statistics of historical exposure measurements for styrene (*n* = 160) during the FRP lamination (spraying and coating) and inspection situations by company.

Exposure Situation (Task)	Company	Product	Task Duration (min)	*n*	AM (ppm)	GM (ppm)	GSD	Min–Max (ppm)	KOEL ^1^ (8 h-TWA)	Analytical Method
FRP lamination (spraying and coating)	A	Ship	180	8	16.49	11.78	2.39	3.77–40.04	20 ppm	NIOSH 1501
	B	Ship	120	2	25.29	19.07	3.06	8.68–41.89		
	C	Ship	60	1	3.10	3.10	-	3.10		
	D	Ship	180	2	25.66	25.64	1.05	24.86–26.45		
	E	Ship	60	4	9.08	8.02	1.74	4.88–17.38		
	F	Ship	180	15	16.44	11.81	2.30	4.25–53.60		
	G	Ship	180	3	10.85	9.67	1.77	6.14–18.41		
	H	Ship	180	1	32.19	32.19	-	32.19		
	I	Ship	180	5	27.27	15.74	4.29	1.67–45.15		
	J	Ship	180	16	19.68	14.77	2.45	1.82–43.67		
	K	Automotive products and repair parts	120	9	19.07	11.97	3.09	2.28–46.45		
	L	Automotive products and repair parts	180	20	49.72	26.06	3.89	1.07–186.34		
	M	Automotive products and repair parts	180	6	13.02	9.27	2.65	2.11–30.21		
	N	Double-walled underground storage tank (UST)	180	1	34.88	34.88	-	34.88		
	O	Double-walled underground storage tank (UST)	180	4	17.93	10.27	3.54	3.52–45.90		
	P	Double-walled underground storage tank (UST)	180	14	17.21	11.04	2.95	1.68–36.60		
	Q	Double-walled underground storage tank (UST)	180	3	12.56	11.51	1.71	6.36–17.95		
	R	Double-walled underground storage tank (UST)	120	4	9.89	7.66	2.66	1.80–15.00		
	S	Sluice	120	10	11.16	7.59	2.56	2.31–37.51		
	T	Sluice	120	4	15.98	9.76	3.06	4.10–43.52		
	U	Sluice	60	1	3.12	3.12	-	3.12		
	V	Double-walled underground storage tank (UST)	180	4	25.85	25.54	1.20	19.52–28.99		
	Total			137	21.80	13.07	2.86	1.07–186.34		
Inspection	A	Ship	60	1	0.07	0.07	-	0.07		
	B	Ship	60	1	0.14	0.14	-	0.14		
	E	Ship	60	1	0.95	0.95	-	0.95		
	G	Ship	60	1	0.01	0.01	-	0.01		
	H	Ship	60	1	0.32	0.32	-	0.32		
	J	Ship	60	1	0.16	0.16	-	0.16		
	L	Automotive products and repair parts	60	6	0.58	0.27	5.84	0.02–1.43		
	M	Automotive products and repair parts	60	3	0.64	0.54	2.14	0.23–0.99		
	N	Double-walled underground storage tank (UST)	60	2	0.04	0.03	1.12	0.03–0.04		
	O	Double-walled underground storage tank (UST)	120	1	0.14	0.14	-	0.14		
	R	Double-walled underground storage tank (UST)	60	2	0.46	0.46	1.02	0.45–0.46		
	S	Sluice	60	1	1.03	1.03	-	1.03		
	T	Sluice	60	1	0.86	0.86	-	0.86		
	V	Double-walled underground storage tank (UST)	60	1	0.28	0.28	-	0.28		
	Total			23	0.45	0.23	4.25	0.01–1.43		

AM: arithmetic mean; GM: geometric mean; GSD: geometric standard deviation; 8 h-TWA: 8-h time weighted average; ^1^ MoEL’s Korean occupational exposure limit (KOEL).

**Table 5:** Comparison of historical exposure measurements with the model estimations of Stoffenmanager and exposure scores calculated using a new model by company.

Exposure Situation (Task)	Company	Product	*n*	Exposure Measurement		Model Estimation				
				GM (ppm)	90th ^1^ (ppm)	Stoffenmanager			New Model	
						Daily Average (ppm)	50th (ppm)	90th (ppm)	Score ^2^	Exposure Category
FRP lamination (spraying and coating)	A	Ship	8	11.78	36.02	1.57	0.46	4.20	6.32	3
	B	Ship	2	19.07	79.86	10.49	4.65	42.02	7.23	3
	C	Ship	1	3.10	-	9.93	8.80	79.35	7.17	3
	D	Ship	2	25.64	27.32	15.73	4.65	42.02	7.86	3
	E	Ship	4	8.02	16.25	0.85	0.76	6.82	6.25	3
	F	Ship	15	11.81	34.39	28.41	8.37	75.59	8.04	3
	G	Ship	3	9.67	20.07	15.73	4.65	42.02	7.17	3
	H	Ship	1	32.19	-	28.64	8.48	76.53	8.78	3
	I	Ship	5	15.74	101.93	29.81	8.80	79.35	8.04	3
	J	Ship	16	14.77	46.55	15.73	4.65	42.02	7.35	3
	K	Automotive products and repair parts	9	11.97	50.93	20.08	8.90	80.29	8.74	3
	L	Automotive products and repair parts	20	26.06	148.93	65.03	19.17	173.02	10.57	4
	M	Automotive products and repair parts	6	9.27	32.32	15.22	4.50	40.61	7.86	3
	N	Double-walled underground storage tank (UST)	1	34.88	-	29.11	8.59	77.47	8.78	3
	O	Double-walled underground storage tank (UST)	4	10.27	51.82	30.99	9.14	82.63	8.74	3
	P	Double-walled underground storage tank (UST)	14	11.04	44.20	27.94	8.26	74.42	8.74	3
	Q	Double-walled underground storage tank (UST)	3	11.51	22.87	32.63	9.62	86.86	8.74	3
	R	Double-walled underground storage tank (UST)	4	7.66	26.88	17.05	7.55	68.31	7.45	3
	S	Sluice	10	7.59	25.31	1.84	0.81	7.35	6.72	3
	T	Sluice	4	9.76	40.85	19.86	8.80	79.35	7.23	3
	U	Sluice	1	3.12	-	9.19	8.14	73.48	7.17	3
	V	Double-walled underground storage tank (UST)	4	25.54	32.36	1.57	0.46	4.18	7.92	3
Inspection	A	Ship	1	0.07	-	0.01	0.01	0.10	2.93	2
	B	Ship	1	0.14	-	0.01	0.01	0.10	3.84	2
	E	Ship	1	0.95	-	0.02	0.02	0.17	3.84	2
	G	Ship	1	0.01	-	0.02	0.02	0.17	4.07	2
	H	Ship	1	0.32	-	0.23	0.21	1.86	4.76	2
	J	Ship	1	0.16	-	0.13	0.11	1.02	3.84	2
	L	Automotive products and repair parts	6	0.27	2.61	0.07	0.06	0.55	4.94	3
	M	Automotive products and repair parts	3	0.54	1.43	0.20	0.18	1.62	4.76	2
	N	Double-walled underground storage tank (UST)	2	0.03	0.04	0.23	0.21	1.89	4.76	2
	O	Double-walled underground storage tank (UST)	1	0.14	-	0.08	0.04	0.33	4.60	2
	R	Double-walled underground storage tank (UST)	2	0.46	0.47	0.21	0.18	1.66	4.76	2
	S	Sluice	1	1.03	-	0.02	0.02	0.18	3.62	2
	T	Sluice	1	0.86	-	0.24	0.21	1.93	4.54	2
	V	Double-walled underground storage tank (UST)	1	0.28	-	0.02	0.02	0.17	3.62	2

^1^ 90th percentiles of the distributions of exposure measurements are calculated using an equation, X_0.90_ = GM × GSD^1.282^; ^2^ All exposure scores calculated using a new model are already log-transformed.

**Table 6:** Bias, relative bias, precision, and accuracy for historical exposure measurements and the model estimations using Stoffenmanager (50th and 90th percentiles).

Exposure Situation (Task)	*n*	Stoffenmanager							
		50th				90th			
		Bias	Relative Bias (%)	Precision	Accuracy	Bias	Relative Bias (%)	Precision	Accuracy
Overall	160	−1.00	−63.32	2.07	1.28	−0.32	−27.30	2.32	1.16
FRP lamination	137	−0.81	−55.60	1.26	1.02	0.06	6.38	0.96	0.75
Inspection	23	−1.30	−72.82	1.64	1.69	−0.92	−60.03	2.12	1.80

## Supplementary Materials

The following are available online at https://www.mdpi.com/1660-4601/17/12/4486/s1, Table S1: The input parameters and model estimations using Stoffenmanager version 8.2, Table S2: The input parameters and calculated exposure scores using a new semi-quantitative model.
