# Supplementary material for: Evaluation of Stoffenmanager and a New Exposure Model for Estimating Occupational Exposure to Styrene in the Fiberglass Reinforced Plastics Lamination Process
Source: Int J Environ Res Public Health. 2020 Jun 22;17(12):4486. doi: 10.3390/ijerph17124486 (PMC7344974; doi:10.3390/ijerph17124486)
Supplement: Supplementary file 1 [file ijerph-17-04486-s001.pdf]

Supplementary Table S1. The input parameters and model estimations using Stoffenmanager version 8.2

No	Exposure situation	Substance	Percentage in the solution	CAS no.	MW	Vapour Pressure (pa) at 25°C	Temperature of the liquid in the process (°C)	Physical state	Type of task	PROC	Duration task (min)	Frequency task	Distance to the task (<1m)	More than 1 employee carrying out the same task?	Followed by a period of drying or curing?	PPE	Name of workplace	Volume of working room	Type of ventilation	Cleaned daily?	Maintenance monthly?	Control measures	Situated in a cabin?	Daily average concentration (mg/m3)	Daily average concentration (ppm)	50th Daily concentration (mg/m3)	50th Daily concentration (ppm)	90th Daily concentration (mg/m3)	90th Daily concentration (ppm)	95th Daily concentration (mg/m3)	95th Daily concentration (ppm)
1	a1_FRP spraying	styrene	44%	100-42-5	104.15	853	25	Liquid	Handling of liquids on large surfaces or large workpieces	PROC7: Industrial spraying	180	4~5 days a week	yes	yes	yes	Half mask respirator with gas/vapour filter	Ship	>1000 m3	general ventilation (open windows and doors)	yes	yes	LEV	Not in a cabin	6.70	1.57	1.98	0.46	17.87	4.20	33.46	7.85
2	a2_Inspection	styrene	44%	100-42-5	104.15	853	25	Liquid	Handling of liquids in negligible amounts	PROC0: Other	60	2~3 days a week	no	no	no	Half mask respirator with gas/vapour filter	Ship	>1000 m3	general ventilation (open windows and doors)	yes	yes	LEV	Not in a cabin	0.05	0.01	0.05	0.01	0.43	0.10	0.81	0.19
3	b1_FRP spraying	styrene	44%	100-42-5	104.15	853	24	Liquid	Handling of liquids on large surfaces or large workpieces	PROC7: Industrial spraying	120	2~3 days a week	yes	yes	yes	no protection	Ship	>1000 m3	general ventilation (open windows and doors)	yes	yes	LEV	Not in a cabin	44.67	10.49	19.79	4.65	179	42.02	335	78.64
4	b2_Inspection	styrene	44%	100-42-5	104.15	853	24	Liquid	Handling of liquids in negligible amounts	PROC0: Other	60	2~3 days a week	no	no	no	no protection	Ship	>1000 m3	general ventilation (open windows and doors)	yes	yes	LEV	Not in a cabin	0.05	0.01	0.05	0.01	0.43	0.10	0.81	0.19
5	c1_FRP spraying	styrene	44%	100-42-5	104.15	853	25	Liquid	Handling of liquids on large surfaces or large workpieces	PROC7: Industrial spraying	60	2~3 days a week	yes	yes	yes	no protection	Ship	>1000 m3	general ventilation (mechanical)	yes	yes	No control measures	Not in a cabin	42.31	9.93	37.49	8.80	338	79.35	634	148.84
6	d1_FRP spraying	styrene	44%	100-42-5	104.15	853	23	Liquid	Handling of liquids (using low pressure but high speed) without creating a mist or spray	PROC7: Industrial spraying	180	4~5 days a week	yes	yes	yes	no protection	Ship	>1000 m3	general ventilation (open windows and doors)	yes	yes	LEV	Not in a cabin	67.00	15.73	19.79	4.65	179	42.02	335	78.64
7	e1_FRP spraying	styrene	33%	100-42-5	104.15	853	24	Liquid	Handling of liquids on large surfaces or large workpieces	PROC7: Industrial spraying	60	2~3 days a week	yes	yes	yes	Half mask respirator with gas/vapour filter	Ship	>1000 m3	general ventilation (mechanical)	yes	yes	No control measures	Not in a cabin	3.63	0.85	3.22	0.76	29.05	6.82	54.41	12.77
8	e2_Inspection	styrene	33%	100-42-5	104.15	853	24	Liquid	Handling of liquids in negligible amounts	PROC0: Other	60	2~3 days a week	no	no	no	Half mask respirator with gas/vapour filter	Ship	>1000 m3	general ventilation (mechanical)	yes	yes	No control measures	Not in a cabin	0.09	0.02	0.08	0.02	0.71	0.17	1.32	0.31
9	f1_FRP spraying	styrene	40%	100-42-5	104.15	853	25	Liquid	Handling of liquids (using low pressure but high speed) without creating a mist or spray	PROC7: Industrial spraying	180	4~5 days a week	yes	yes	yes	no protection	Ship	>1000 m3	general ventilation (open windows and doors)	yes	yes	No control measures	Not in a cabin	121.00	28.41	35.64	8.37	322	75.59	603	141.56
10	g1_FRP spraying	styrene	44%	100-42-5	104.15	853	25	Liquid	Handling of liquids (using low pressure but high speed) without creating a mist or spray	PROC7: Industrial spraying	180	4~5 days a week	yes	yes	yes	no protection	Ship	>1000 m3	general ventilation (mechanical)	yes	yes	LEV	Not in a cabin	67.00	15.73	19.79	4.65	179	42.02	335	78.64
11	g2_Inspection	styrene	44%	100-42-5	104.15	853	25	Liquid	Handling of liquids in negligible amounts	PROC0: Other	60	2~3 days a week	no	no	no	no protection	Ship	>1000 m3	general ventilation (mechanical)	yes	yes	LEV	Not in a cabin	0.09	0.02	0.08	0.02	0.71	0.17	1.32	0.31
12	h1_FRP spraying	styrene	41%	100-42-5	104.15	853	23	Liquid	Handling of liquids on large surfaces or large workpieces	PROC7: Industrial spraying	180	4~5 days a week	yes	yes	yes	no protection	Ship	>1000 m3	general ventilation (mechanical)	yes	yes	No control measures	Not in a cabin	122.00	28.64	36.11	8.48	326	76.53	611	143.44
13	h2_Inspection	styrene	41%	100-42-5	104.15	853	23	Liquid	Handling of liquids in negligible amounts	PROC0: Other	60	2~3 days a week	no	no	no	no protection	Ship	>1000 m3	general ventilation (mechanical)	yes	yes	No control measures	Not in a cabin	0.99	0.23	0.88	0.21	7.93	1.86	14.86	3.49
14	i1_FRP spraying	styrene	44%	100-42-5	104.15	853	24	Liquid	Handling of liquids (using low pressure but high speed) without creating a mist or spray	PROC7: Industrial spraying	180	4~5 days a week	yes	yes	yes	no protection	Ship	>1000 m3	general ventilation (open windows and doors)	yes	yes	No control measures	Not in a cabin	127.00	29.81	37.49	8.80	338	79.35	634	148.84
15	j1_FRP spraying	styrene	44%	100-42-5	104.15	853	25	Liquid	Handling of liquids (using low pressure but high speed) without creating a mist or spray	PROC7: Industrial spraying	180	4~5 days a week	yes	yes	yes	no protection	Ship	>1000 m3	general ventilation (open windows and doors)	yes	yes	LEV	Not in a cabin	67.00	15.73	19.79	4.65	179	42.02	335	78.64
16	j2_Inspection	styrene	44%	100-42-5	104.15	853	25	Liquid	Handling of liquids in negligible amounts	PROC0: Other	60	2~3 days a week	no	no	no	no protection	Ship	>1000 m3	general ventilation (open windows and doors)	yes	yes	LEV	Not in a cabin	0.54	0.13	0.48	0.11	4.35	1.02	8.14	1.91
17	k1_FRP spraying	styrene	38%	100-42-5	104.15	853	24	Liquid	Handling of liquids on large surfaces or large workpieces	PROC7: Industrial spraying	120	4~5 days a week	yes	yes	yes	no protection	Motor vehicle and parts	100-1000 m3	general ventilation (open windows and doors)	yes	yes	No control measures	Not in a cabin	85.54	20.08	37.90	8.90	342	80.29	641	150.48
18	L1_FRP spraying	styrene	35%	100-42-5	104.15	853	25	Liquid	Handling of liquids on large surfaces or large workpieces	PROC7: Industrial spraying	180	4~5 days a week	yes	yes	yes	no protection	Motor vehicle and parts	<100 m3	general ventilation (open windows and doors)	yes	yes	No control measures	Not in a cabin	277.00	65.03	81.68	19.17	737	173.02	1381	324.20
19	L2_Inspection	styrene	35%	100-42-5	104.15	853	25	Liquid	Handling of liquids in negligible amounts	PROC0: Other	60	2~3 days a week	no	no	no	Half mask respirator with gas/vapour filter	Motor vehicle and parts	<100 m3	general ventilation (open windows and doors)	yes	yes	LEV	Not in a cabin	0.29	0.07	0.26	0.06	2.34	0.55	4.38	1.03
20	m1_FRP spraying	styrene	35%	100-42-5	104.15	853	24	Liquid	Handling of liquids on large surfaces or large workpieces	PROC7: Industrial spraying	180	4~5 days a week	yes	yes	yes	no protection	Motor vehicle and parts	100-1000 m3	general ventilation (mechanical)	yes	yes	LEV	Not in a cabin	64.84	15.22	19.15	4.50	173	40.61	324	76.06
21	m2_Inspection	styrene	35%	100-42-5	104.15	853	24	Liquid	Handling of liquids in negligible amounts	PROC0: Other	60	2~3 days a week	no	no	no	no protection	Motor vehicle and parts	100-1000 m3	general ventilation (mechanical)	yes	yes	LEV	Not in a cabin	0.86	0.20	0.76	0.18	6.9	1.62	12.92	3.03
22	n1_FRP spraying	styrene	42%	100-42-5	104.15	853	25	Liquid	Handling of liquids on large surfaces or large workpieces	PROC7: Industrial spraying	180	4~5 days a week	yes	yes	yes	no protection	Underground storage tank (UST)	>1000 m3	general ventilation (mechanical)	yes	yes	No control measures	Not in a cabin	124.00	29.11	36.58	8.59	330	77.47	618	145.08
23	n2_Inspection	styrene	42%	100-42-5	104.15	853	25	Liquid	Handling of liquids in negligible amounts	PROC0: Other	60	2~3 days a week	no	no	no	no protection	Underground storage tank (UST)	>1000 m3	general ventilation (mechanical)	yes	yes	No control measures	Not in a cabin	1.00	0.23	0.89	0.21	8.04	1.89	15.05	3.53
24	o1_FRP spraying	styrene	40%	100-42-5	104.15	853	23	Liquid	Handling of liquids on large surfaces or large workpieces	PROC7: Industrial spraying	180	4~5 days a week	yes	yes	yes	no protection	Underground storage tank (UST)	100-1000 m3	general ventilation (open windows and doors)	yes	yes	No control measures	Not in a cabin	132.00	30.99	38.95	9.14	352	82.63	658	154.47
25	o2_Inspection	styrene	40%	100-42-5	104.15	853	23	Liquid	Handling of liquids in negligible amounts	PROC0: Other	120	2~3 days a week	no	no	no	Half mask respirator with gas/vapour filter	Underground storage tank (UST)	100-1000 m3	general ventilation (open windows and doors)	yes	yes	No control measures	Not in a cabin	0.35	0.08	0.16	0.04	1.40	0.33	2.63	0.62
26	p1_FRP spraying	styrene	33%	100-42-5	104.15	853	25	Liquid	Handling of liquids on large surfaces or large workpieces	PROC7: Industrial spraying	180	4~5 days a week	yes	yes	yes	no protection	Underground storage tank (UST)	100-1000 m3	general ventilation (open windows and doors)	yes	yes	No control measures	Not in a cabin	119.00	27.94	35.17	8.26	317	74.42	595	139.68
27	q1_FRP spraying	styrene	44%	100-42-5	104.15	853	24	Liquid	Handling of liquids on large surfaces or large workpieces	PROC7: Industrial spraying	180	4~5 days a week	yes	yes	yes	no protection	Underground storage tank (UST)	100-1000 m3	general ventilation (open windows and doors)	yes	yes	No control measures	Not in a cabin	139.00	32.63	40.97	9.62	370	86.86	693	162.69
28	r1_FRP spraying	styrene	33%	100-42-5	104.15	853	25	Liquid	Handling of liquids on large surfaces or large workpieces	PROC7: Industrial spraying	120	2~3 days a week	yes	yes	yes	no protection	Underground storage tank (UST)	>1000 m3	general ventilation (mechanical)	yes	yes	No control measures	Not in a cabin	72.64	17.05	32.18	7.55	291	68.31	544	127.71
29	r2_Inspection	styrene	33%	100-42-5	104.15	853	25	Liquid	Handling of liquids in negligible amounts	PROC0: Other	60	2~3 days a week	no	no	no	no protection	Underground storage tank (UST)	>1000 m3	general ventilation (mechanical)	yes	yes	No control measures	Not in a cabin	0.88	0.21	0.78	0.18	7.07	1.66	13.24	3.11
30	s1_FRP spraying	styrene	38%	100-42-5	104.15	853	24	Liquid	Handling of liquids on large surfaces or large workpieces	PROC7: Industrial spraying	120	4~5 days a week	yes	yes	yes	Half mask respirator with gas/vapour filter	Sluice	>1000 m3	general ventilation (open windows and doors)	yes	yes	No control measures	Not in a cabin	7.83	1.84	3.47	0.81	31.31	7.35	58.64	13.77
31	s2_Inspection	styrene	38%	100-42-5	104.15	853	24	Liquid	Handling of liquids in negligible amounts	PROC0: Other	60	2~3 days a week	no	no	no	Half mask respirator with gas/vapour filter	Sluice	>1000 m3	general ventilation (open windows and doors)	yes	yes	No control measures	Not in a cabin	0.10	0.02	0.08	0.02	0.76	0.18	1.43	0.34
32	t1_FRP spraying	styrene	44%	100-42-5	104.15	853	25	Liquid	Handling of liquids on large surfaces or large workpieces	PROC7: Industrial spraying	120	2~3 days a week	yes	yes	yes	no protection	Sluice	>1000 m3	general ventilation (open windows and doors)	yes	yes	No control measures	Not in a cabin	84.61	19.86	37.49	8.80	338	79.35	634	148.84
33	t2_Inspection	styrene	44%	100-42-5	104.15	853	25	Liquid	Handling of liquids in negligible amounts	PROC0: Other	60	2~3 days a week	no	no	no	no protection	Sluice	>1000 m3	general ventilation (open windows and doors)	yes	yes	No control measures	Not in a cabin	1.03	0.24	0.91	0.21	8.24	1.93	15.42	3.62
34	u1_FRP spraying	styrene	38%	100-42-5	104.15	853	23	Liquid	Handling of liquids on large surfaces or large workpieces	PROC7: Industrial spraying	60	2~3 days a week	yes	yes	yes	no protection	Sluice	>1000 m3	general ventilation (mechanical)	yes	yes	No control measures	Not in a cabin	39.14	9.19	34.69	8.14	313	73.48	586	137.57
35	v1_FRP spraying	styrene	37%	100-42-5	104.15	853	24	Liquid	Handling of liquids on large surfaces or large workpieces	PROC7: Industrial spraying	180	4~5 days a week	yes	yes	yes	Half mask respirator with gas/vapour filter	Underground storage tank (UST)	100-1000 m3	general ventilation (open windows and doors)	yes	yes	LEV	Not in a cabin	6.68	1.57	1.97	0.46	17.81	4.18	33.35	7.83
36	v2_Inspection	styrene	37%	100-42-5	104.15	853	24	Liquid	Handling of liquids in negligible amounts	PROC0: Other	60	2~3 days a week	no	no	no	Half mask respirator with gas/vapour filter	Underground storage tank (UST)	100-1000 m3	general ventilation (open windows and doors)	yes	yes	LEV	Not in a cabin	0.09	0.02	0.08	0.02	0.71	0.17	1.33	0.31

Supplementary Table S2. The input parameters and calculated exposure scores using a new semi-quantitative model

No	Exposure situation	Substance	Percentage in the solution	CAS no.	MW	Vapour Pressure (pa) at 25°C	Temperature of the liquid in the process (◦C)	Name of workplace	Position factor (Pf)	Potential emission and handing of products (Ep)	Historical exposure (Eh)	Type of process with tool cleaning, inspection, and maintenance (Em)	Ventilation and containment (η_{gv})	Localized control measure (Lc)	Personal protective equipment (PPE)	Health hazard category (H)	Task duration (Th)	Task frequency (Tf)	Distance from the source (D)	Room volume (V)	Exposure score	Log-transformed exposure score	Exposure category
1	a1_FRP spraying	styrene	44%	100-42-5	104.15	853	25	Ship	2.0	3.0	2.0	2.0	1.2	2.0	2.0	4.0	2.0	3.0	2.0	10.0	553.0	6.32	3
2	a2_Inspection	styrene	44%	100-42-5	104.15	853	25	Ship	1.2	1.0	1.2	2.0	1.2	1.5	2.0	4.0	1.5	2.0	1.5	10.0	18.7	2.93	2
3	b1_FRP spraying	styrene	44%	100-42-5	104.15	853	24	Ship	2.0	3.0	3.0	2.0	1.2	2.0	5.0	4.0	2.0	2.0	2.0	10.0	1382.4	7.23	3
4	b2_Inspection	styrene	44%	100-42-5	104.15	853	24	Ship	1.2	1.0	1.2	2.0	1.2	1.5	5.0	4.0	1.5	2.0	1.5	10.0	46.7	3.84	2
5	c1_FRP spraying	styrene	44%	100-42-5	104.15	853	25	Ship	2.0	3.0	2.0	2.0	1.5	3.0	5.0	4.0	1.5	2.0	2.0	10.0	1296.0	7.17	3
6	d1_FRP spraying	styrene	44%	100-42-5	104.15	853	23	Ship	2.0	3.0	5.0	2.0	1.2	1.5	5.0	4.0	2.0	3.0	2.0	10.0	2592.0	7.86	3
7	e1_FRP spraying	styrene	33%	100-42-5	104.15	853	24	Ship	2.0	3.0	2.0	2.0	1.5	3.0	2.0	4.0	1.5	2.0	2.0	10.0	518.4	6.25	3
8	e2_Inspection	styrene	33%	100-42-5	104.15	853	24	Ship	1.2	1.0	1.2	2.0	1.5	3.0	2.0	4.0	1.5	2.0	1.5	10.0	46.7	3.84	2
9	f1_FRP spraying	styrene	40%	100-42-5	104.15	853	25	Ship	2.0	3.0	3.0	2.0	1.2	3.0	5.0	4.0	2.0	3.0	2.0	10.0	3110.4	8.04	3
10	g1_FRP spraying	styrene	44%	100-42-5	104.15	853	25	Ship	2.0	3.0	2.0	2.0	1.5	1.5	5.0	4.0	2.0	3.0	2.0	10.0	1296.0	7.17	3
11	g2_Inspection	styrene	44%	100-42-5	104.15	853	25	Ship	1.2	1.0	1.2	2.0	1.5	1.5	5.0	4.0	1.5	2.0	1.5	10.0	58.3	4.07	2
12	h1_FRP spraying	styrene	41%	100-42-5	104.15	853	23	Ship	2.0	3.0	5.0	2.0	1.5	3.0	5.0	4.0	2.0	3.0	2.0	10.0	6480.0	8.78	3
13	h2_Inspection	styrene	41%	100-42-5	104.15	853	23	Ship	1.2	1.0	1.2	2.0	1.5	3.0	5.0	4.0	1.5	2.0	1.5	10.0	116.6	4.76	2
14	i1_FRP spraying	styrene	44%	100-42-5	104.15	853	24	Ship	2.0	3.0	3.0	2.0	1.2	3.0	5.0	4.0	2.0	3.0	2.0	10.0	3110.4	8.04	3
15	j1_FRP spraying	styrene	44%	100-42-5	104.15	853	25	Ship	2.0	3.0	3.0	2.0	1.2	1.5	5.0	4.0	2.0	3.0	2.0	10.0	1555.2	7.35	3
16	j2_Inspection	styrene	44%	100-42-5	104.15	853	25	Ship	1.2	1.0	1.2	2.0	1.2	1.5	5.0	4.0	1.5	2.0	1.5	10.0	46.7	3.84	2
17	k1_FRP spraying	styrene	38%	100-42-5	104.15	853	24	Motor vehicle and parts	2.0	3.0	3.0	2.0	1.2	3.0	5.0	4.0	2.0	3.0	2.0	5.0	6220.8	8.74	3
18	L1_FRP spraying	styrene	35%	100-42-5	104.15	853	25	Motor vehicle and parts	2.0	3.0	5.0	1.5	1.2	3.0	5.0	4.0	2.0	3.0	2.0	1.0	38880.0	10.57	4
19	L2_Inspection	styrene	35%	100-42-5	104.15	853	25	Motor vehicle and parts	1.2	1.0	1.2	1.5	1.2	1.5	2.0	4.0	1.5	2.0	1.5	1.0	140.0	4.94	3
20	m1_FRP spraying	styrene	35%	100-42-5	104.15	853	24	Motor vehicle and parts	2.0	3.0	2.0	2.0	1.5	1.5	5.0	4.0	2.0	3.0	2.0	5.0	2592.0	7.86	3
21	m2_Inspection	styrene	35%	100-42-5	104.15	853	24	Motor vehicle and parts	1.2	1.0	1.2	2.0	1.5	1.5	5.0	4.0	1.5	2.0	1.5	5.0	116.6	4.76	2
22	n1_FRP spraying	styrene	42%	100-42-5	104.15	853	25	Underground storage tank (UST)	2.0	3.0	5.0	2.0	1.5	3.0	5.0	4.0	2.0	3.0	2.0	10.0	6480.0	8.78	3
23	n2_Inspection	styrene	42%	100-42-5	104.15	853	25	Underground storage tank (UST)	1.2	1.0	1.2	2.0	1.5	3.0	5.0	4.0	1.5	2.0	1.5	10.0	116.6	4.76	2
24	o1_FRP spraying	styrene	40%	100-42-5	104.15	853	23	Underground storage tank (UST)	2.0	3.0	3.0	2.0	1.2	3.0	5.0	4.0	2.0	3.0	2.0	5.0	6220.8	8.74	3
25	o2_Inspection	styrene	40%	100-42-5	104.15	853	23	Underground storage tank (UST)	1.2	1.0	1.2	2.0	1.2	3.0	2.0	4.0	2.0	2.0	1.5	5.0	99.5	4.60	2
26	p1_FRP spraying	styrene	33%	100-42-5	104.15	853	25	Underground storage tank (UST)	2.0	3.0	3.0	2.0	1.2	3.0	5.0	4.0	2.0	3.0	2.0	5.0	6220.8	8.74	3
27	q1_FRP spraying	styrene	44%	100-42-5	104.15	853	24	Underground storage tank (UST)	2.0	3.0	3.0	2.0	1.2	3.0	5.0	4.0	2.0	3.0	2.0	5.0	6220.8	8.74	3
28	r1_FRP spraying	styrene	33%	100-42-5	104.15	853	25	Underground storage tank (UST)	2.0	3.0	2.0	2.0	1.5	3.0	5.0	4.0	2.0	2.0	2.0	10.0	1728.0	7.45	3
29	r2_Inspection	styrene	33%	100-42-5	104.15	853	25	Underground storage tank (UST)	1.2	1.0	1.2	2.0	1.5	3.0	5.0	4.0	1.5	2.0	1.5	10.0	116.6	4.76	2
30	s1_FRP spraying	styrene	38%	100-42-5	104.15	853	24	Sluice	2.0	3.0	2.0	2.0	1.2	3.0	2.0	4.0	2.0	3.0	2.0	10.0	829.4	6.72	3
31	s2_Inspection	styrene	38%	100-42-5	104.15	853	24	Sluice	1.2	1.0	1.2	2.0	1.2	3.0	2.0	4.0	1.5	2.0	1.5	10.0	37.3	3.62	2
32	t1_FRP spraying	styrene	44%	100-42-5	104.15	853	25	Sluice	2.0	3.0	2.0	2.0	1.2	3.0	5.0	4.0	2.0	2.0	2.0	10.0	1382.4	7.23	3
33	t2_Inspection	styrene	44%	100-42-5	104.15	853	25	Sluice	1.2	1.0	1.2	2.0	1.2	3.0	5.0	4.0	1.5	2.0	1.5	10.0	93.3	4.54	2
34	u1_FRP spraying	styrene	38%	100-42-5	104.15	853	23	Sluice	2.0	3.0	2.0	2.0	1.5	3.0	5.0	4.0	1.5	2.0	2.0	10.0	1296.0	7.17	3
35	v1_FRP spraying	styrene	37%	100-42-5	104.15	853	24	Underground storage tank (UST)	2.0	3.0	5.0	2.0	1.2	2.0	2.0	4.0	2.0	3.0	2.0	5.0	2764.8	7.92	3
36	v2_Inspection	styrene	37%	100-42-5	104.15	853	24	Underground storage tank (UST)	1.2	1.0	1.2	2.0	1.2	1.5	2.0	4.0	1.5	2.0	1.5	5.0	37.3	3.62	2
